# NPY Receptor 2 Mediates NPY Antidepressant Effect in the mPFC of LPS Rat by Suppressing NLRP3 Signaling Pathway

**DOI:** 10.1155/2019/7898095

**Published:** 2019-05-15

**Authors:** Wenjiao Wang, Tao Xu, Xinyue Chen, Kemeng Dong, Chunkai Du, Jing Sun, Cuige Shi, Xiaoxiao Li, Yutao Yang, Hui Li, Zhi-Qing David Xu

**Affiliations:** ^1^Department of Neurobiology, Beijing Key Laboratory of Neural Regeneration and Repair, Beijing Laboratory of Brain Disorders (Ministry of Science and Technology), Beijing Institute of Brain Disorders, Capital Medical University, Beijing, China; ^2^Department of Anatomy, Capital Medical University, Beijing, China; ^3^Department of Pathology, Capital Medical University, Beijing, China; ^4^Department of Cell Biology, National Research Institute of Family Planning, Beijing, China

## Abstract

Accumulated evidences show that neuroinflammation play a pivotal role in the pathogenesis of depression. Neuropeptide Y (NPY) and its receptors have been demonstrated to have anti-inflammative as well as antidepressant effects. In the present study, the ability of NPY to modulate depressive-like behaviors induced by lipopolysaccharides (LPS) in rats and the receptors and signaling mechanisms involved were investigated. Continuous injection LPS (i.p) for 4 days led to development of depressive-like behaviors in rats, accompanied with M1-type microglia activation and increased levels of IL-1*β* as well as decreased levels of NPY and Y2R expression in the mPFC selectively. Local injection of NPY into the medial prefrontal cortex (mPFC) ameliorated the depression-like behaviors and suppressed the NLRP3 inflammasome signaling pathway. Y2R agonist PYY (3-36) mimicked and Y2R antagonist BIIE0246 abolished the NPY effects in the mPFC. All these results suggest that NPY and Y2R in the mPFC are involved in the pathophysiology of depression and NPY plays an antidepressant role in the mPFC mainly via Y2R, which suppresses the NLRP3 signaling pathway, in LPS-induced depression model rats.

## 1. Introduction

Inflammasome activation in the central nervous system (CNS) and cell-mediated immune response are the prominent feature associated with depression symptom, duration, or severity [1–4]. Studies in postmortem samples of depressed individuals who died by suicide demonstrated that both mRNA and protein levels of IL-1*β*, IL-6, and TNF-*α* are significantly increased, and anti-inflammatory cytokine IL-10 and IL-4 are significantly decreased in the PFC [5]. Major depression disorder (MDD) with antidepressant-resistant patients is also accompanied with increased concentration of IL-1, IL-6, TNF-*α*, and acute phase reactants in plasma compared with treatment-responsive patients [6]. Studies from rattus depression model demonstrated similar results as well [7]. Therefore, prevention of inflammatory disturbances has been acknowledged as a potential avenue for treatment of depression

Neuropeptide Y (NPY) is one of the most abundant peptides in the CNS, which exerts its variety of physiological responses via five receptor subtypes, termed Y1R, Y2R, Y4R, Y5R, and Y6R. NPY and its receptors are widely expressed in brain regions regulating depression and stress resilience, such as cortex, hypothalamus, and hippocampus [8, 9]. Y1R and Y2R are the most abundant receptor types in the CNS [10–12]. Clinical studies showed NPY variant rs16139 and Y2R variant rs6857715 are associated with MDD [13, 14]. Moreover, NPY plays anti-inflammatory actions via Y1/Y2 receptors in the monocytes and granulocytes of the peripheral blood of lipopolysaccharide- (LPS-) induced inflammation rat model [15].

In the present study, we aimed to investigate the ability of NPY to modulate depressive-like behaviors of LPS-treated rats. Moreover, the receptors and signaling mechanisms involved were also investigated.

## 2. Materials and Methods

### 2.1. Animals and Housing

The experiments in this article were performed on adult Sprague-Dawley rats (2 months old, weighing 200-220 g, Beijing Vital River Laboratory Animal Technology Co. Ltd, China). All rats were acclimatized for one week prior to experiment and housed three per standard size cage with food and water available unless special instructions. Animal rooms were maintained a temperature of 20-25°C and a constant light/dark cycle (lights on: 7:00-19:00). The study was approved by the Animal Care Committee at Capital Medical University. Animals were divided into two experimental groups: the control (CTL) group was treated with saline; the LPS group was administered with LPS (Escherichia coli 055: B5, No. L-2880, Sigma-Aldrich, St. Louis, MO, USA), freshly dissolved in sterile saline prior to injection, at a dose of 500 *μ*g/kg. Both the CTL and LPS group rats were injected intraperitoneally between 09:00 and 10:00 a.m. for 4 days. The administered dose and the duration of the treatment were based on a pilot experiment in our lab.

### 2.2. Depressive-Like Behavior Tests

#### 2.2.1. Open-Field Test

Open-Field Test (OFT) was quantified for 5 min in the apparatus consisted of a black square arena (125 × 125 cm) and a 40 cm high opaque black wall. All rats were placed in a testing room 30 min before the test took place in order to allow them to acclimate. Each rat was gently placed in the center of the open-field box. During the test, rat was allowed to explore freely in the open field. The distance of horizontal and vertical activity was videotaped and quantified with NAY-maze. The arena was carefully cleaned after each test.

#### 2.2.2. Sucrose Preference Test

To verify anhedonia, the sucrose preference test (SPT) was carried out as described in our earlier study [16], Briefly, rats were water-deprived for 8 h, then were presented with two preweighed bottles, one contained with 1% sucrose solution, the other contained with tap water. Moreover, the placement of two bottles (left/right) was counterbalanced and interchanged 30 min after the test started. The total time of SPT is 1 h. Sucrose consumption was calculated according to the following formula: sucrose preference = [sucrose intake/(sucrose intake + water intake)] × 100%.

### 2.3. Stereotactical Injection

**Figure 1:**
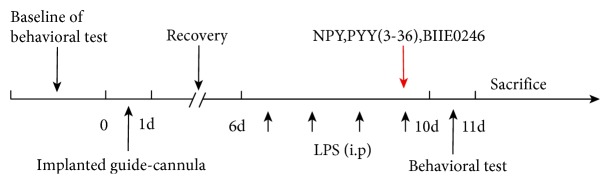
Time schedule for the experiment. LPS was injected for 4 days. Depressive-like behavior tests included OFT and SPT. NPY (1 nmol), Y2R agonist (PYY (3-36), 1 nmol), or Y2R antagonist (BIIE0246, 40 nmol) treatment were locally injected into the mPFC.

Rats were anesthetized with 6% chloral hydrate (6 ml/kg) administrated i.p, then stereotaxically implanted guide cannula (RWD Life Science and Technology, Shenzhen, China) into the bilateral mPFC (the stereotaxic coordinates were −3.2 mm bregma, −0.5 mm lateral, and 4.0 mm below the surface of the skull) according to *The Rat Brain in Stereotactic Coordinates* [17]. The guide cannula were closed by a stylet, then were fixed onto the skull with 3 stainless steel screws and dental cement. After the surgery, rats were allowed a 6-day recovery. To evaluate the effect of NPY and Y2R on depressive-related behaviors within the mPFC, NPY (1 nmol, Bachem, England), PYY (3-36) (Y2R agonist, 1 nmol, Tocris, England), and BIBE0246 (Y2 antagonist, 40 nmol, Tocris, England) were dissolved in 0.9% saline and infused into the bilateral mPFC once after the last injection of LPS. After infusion, the injection tube was left for 5 min. The doses of NPY, PYY (3-36), and BIBE0246 were chosen based on published literatures [18–20]. The experiments were carried out according to the schedule shown in Figure 1.

### 2.4. Quantitative Real-Time PCR Analysis

Brains were rapidly separated from the skull, and the mPFC and ventral hippocampus were removed under RNase-free conditions and immediately frozen in liquid nitrogen and then stored at −80°C for later RNA and protein extraction. The total RNA of the mPFC and ventral hippocampus was extracted using the RNeasy Lipid Tissue Mini Kit (Qiagen, Germany) following the manufacturer's instructions. RNA concentrations were measured using the NanoDrop 2000 (Thermo Fisher Scientific, Wilmington, DE, USA) with 260 nm/280 nm ratios between 1.8 and 2.2. RNA (1 *μ*g) was reverse transcribed into first-strand cDNA by applying the Transcriptor First Strand cDNA Synthesis Kit (Roche, Indianapolis, IN, USA) following the manufacturer's instructions.

Quantitative PCR (Q-PCR) was performed according to previous studies [21]. All assays were run in triplicate, and GAPDH was used as the internal control for each sample. The sequences of the primers used in this study are provided in Table 1. Reaction protocol was 2 min at 60°C and 10 min at 95°C, followed by 40 cycle reactions as 15 s of denaturing at 95°C and 1 min of annealing at 60°C. Samples were held at 10°C at the end of each amplification reaction. The expression levels of target mRNAs are based on the △Ct value.

### 2.5. Western Blot Analysis

Total protein extraction was as described in our previous studies [22]. The information of primary antibody is shown in Table 2. Membranes were washed for three times (10 min × 3) with Tris-buffered saline-Tween (TBST) and incubated with horseradish peroxidase- (HRP-) conjugated secondary antibody (1,5000, Absin, Beijing, China) at room temperature for 1 h then washed three times (10 min×3) with Tris-buffered saline-Tween (TBST). The bands on the membrane were visualized by enhanced chemiluminescence (ImageQuant LAS 500). ImageJ analysis software (NIH, MD, USA) was applied to quantify the signal. Each experiment was repeated three times, and the results were averaged and normalized.

### 2.6. IL-1*β* Assay

Frozen mPFC brain tissue samples were weighed and transferred to tubes on ice containing 10 times volume of test buffer supplied by ELISA kit (EK301B2/2, Multi Science, China). All samples were centrifuged at 3000 rpm (rounds per min) for 10 min. Plasma was collected and precipitation was abandoned. The standard of IL-1*β* supplied by ELISA kit was diluted at concentrations of 2, 1, 0.5, 0.25, 0.125, 0.0625, and 0.0312 ng/ml. Levels of IL-1*β* were measured by ELISA kit based on a standard curve drawn by gradient dilution of the IL-1*β* standard. The absorbance at 450 nm was measured by using an ELISA plate reader (Multiskan MK3, Thermo Fisher Scientific, USA). Results combined with the measured concentration value and weighing value are expressed as pg/mg.

### 2.7. Statistical Analysis

The data in this article were presented as mean ± SEM (standard error of measurements). Statistical analysis was analyzed using the SPSS 19.0 software. Student's *t*-test and one-way ANOVA analysis followed by the LSD multiple comparison tests were selected. *P* < 0.05 was considered statistically significant.

## 3. Results

### 3.1. LPS-Induced Depressive-Like Behaviors in Rats

**Figure 2:**
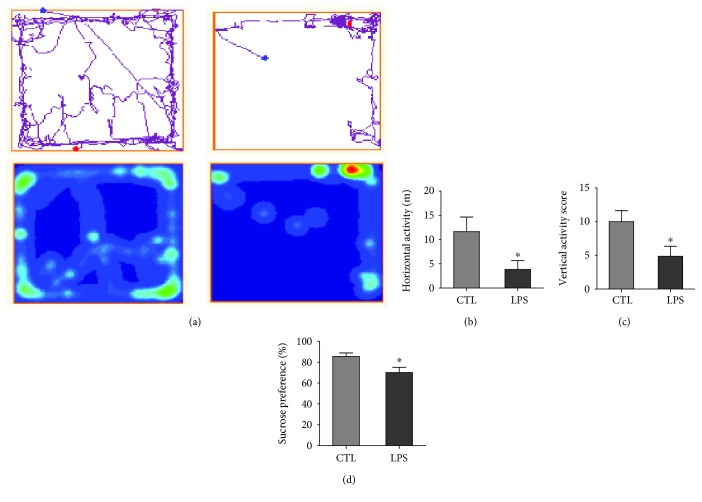
LPS group rats exhibited depressive-like behaviors in SPT and OFT compared with the CTL group. (a) The locomotion track of the CTL and LPS groups. The red area represented the longer residence of the rats, while the green area represents the shorter residence and the blue area represents the least residence of rats. (b, c) LPS group rats showed a lower activity score in OFT compared to the CTL group, horizontal activity score in OFT: ^∗^*P* < 0.05, *t*(10) = 2.202, *P* = 0.0479; vertical activity score in OFT: ^∗^*P* < 0.05, *t*(10) = 2.317, *P* = 0.039. (d) LPS group rats showed a decreased consumption of sucrose in SPT compared to the CTL group, ^∗∗^*P* < 0.01, *t*(10) = 2.611, *P* = 0.0228. CTL: *n* = 6, LPS: *n* = 6. Values are expressed as mean ± SEM. Independent *t*-test results are shown in this figure.

Depressive-like behaviors were assessed on the last day of LPS injection (Figure 1). In OFT, the LPS group rats moved mostly at the edge and rarely at the central area compared with the CTL group in the open-field box (Figure 2(a)). The LPS model rats showed significantly lower horizontal (*P* < 0.05) and vertical scores than the CTL group (*P* < 0.05) (Figures 2(b) and 2(c)). In SPT, the LPS group consumed significantly less sucrose solution than the CTL group (*P* < 0.05) (Figure 2(d)).

### 3.2. M1-Type Microglia and NLRP3 Inflammasome Signaling Were Activated in the mPFC and Ventral Hippocampus of LPS Model Rats

**Figure 3:**
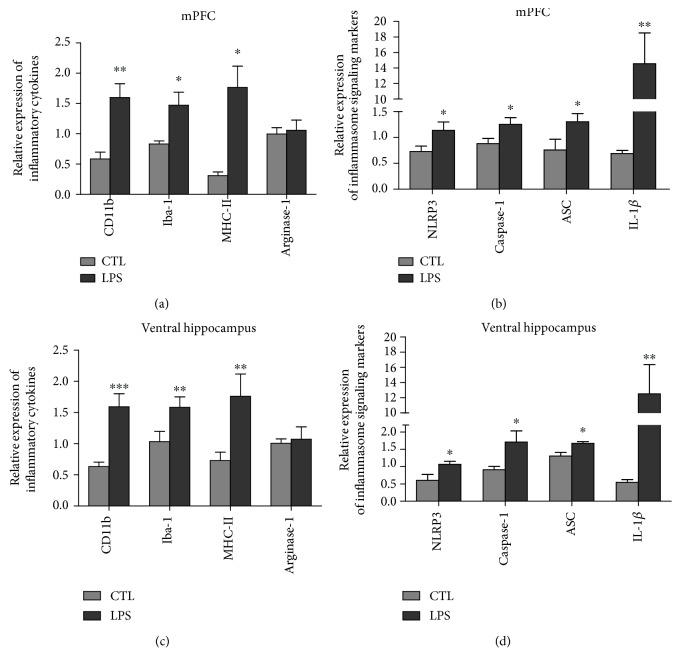
The mRNA expression of M1-type microglia and NLRP3 signaling markers was upregulated in the mPFC and ventral hippocampus of LPS-induced depression rats. (a, c) LPS group rats showed increased mRNA expression of M1-type microglia markers CD11b, Iba-1, and MHC-II compared to CTL rats in the mPFC and ventral hippocampus. In the mPFC: CD11b: *P* < 0.01, *t*(10) = 3.348, *P* = 0.0058; Iba-1: *P* < 0.05, *t*(10) = 2.337, *P* = 0.0376; MHC-II: *P* < 0.05, *t*(10) = 2.721, *P* = 0.0186. In the ventral hippocampus: CD11b: *P* < 0.001, *t*(10) = 4.136, *P* = 0.0007; Iba-1: *P* < 0.01, *t*(10) = 2.921, *P* = 0.0091; MHC-II: *P* < 0.01, *t*(10) = 4.123, *P* = 0.0010. However, there was no significant difference of M2-type microglia marker Arginase-1 between two groups in both the mPFC *P* > 0.05, *t*(10) = 0.3215, *P* = 0.7518 and the ventral hippocampus *P* > 0.05, *t*(10) = 0.2578, *P* = 0.6309 (a, c). (b, d) LPS group rats showed increased mRNA expression of NLRP3 pathway markers NLRP3, caspase-1, ASC, and IL-1*β* compared to CTL rats. In the mPFC: NLRP3: *P* < 0.05, *t*(10) = 2.138, *P* = 0.0473; casepase-1: *P* < 0.05, *t*(10) = 2.276, *P* = 0.0361; ASC, *P* < 0.05, *t*(10) = 2.156, *P* = 0.0421; IL-1*β*: *P* < 0.01, *t*(10) = 3.678, *P* = 0.009. In the ventral hippocampus: NLRP3: *P* < 0.05, *t*(12) = 2.634, *P* = 0.0272; caspase-1: *P* < 0.05, *t*(10) = 2.322, *P* = 0.0322; ASC: *P* < 0.05, *t*(10) = 2.978, *P* = 0.0139; IL-1*β*: *P* < 0.01, *t*(10) = 3.306, *P* = 0.0042. CTL: *n* = 6, LPS: *n* = 6. Values were expressed as mean ± SEM. Data was analyzed by independent *t*-test.

To assess the phenotype of microglia of LPS-induced inflammation in the CNS, the mRNA expression levels of M1-type microglia markers (CD11b, Iba-1, and MHC-II) and M2-type microglia markers (Arginase-1, CD206, and IL10) in the mPFC and ventral hippocampus region of rats were analyzed using Q-PCR. The expression of CD11b, Iba-1, and MHC-II in the mPFC was significantly increased in the LPS group rats compared to the CTL group rats (Table 3, Figure 3(a)). Similar results were also seen in the ventral hippocampus (Table 3, Figure 3(c)). Meanwhile, no significant difference in the mRNA expression levels of Arginase-1 was observed in both the mPFC and the ventral hippocampus between two groups (Table 3, Figures 3(a) and 3(c)). The expression level of CD206 and IL10 was too low to make an appropriate analysis as their CT values were over 35 (data not shown).

To determine whether LPS activates M1 phenotype microglia through the NLPR3 signaling pathway, we examined the mRNA expression levels of the NLRP3 pathway markers including NLRP3, caspase-1, ASC, and IL-1*β* in the mPFC and ventral hippocampus. Thus, NLRP3, caspase-1, ASC, and IL-1*β* mRNA levels were increased in both the mPFC and the ventral hippocampus from LPS rats compared to control rats (Table 3, Figure 3(b)), indicating an involvement of the NLPR3 signaling pathway.

### 3.3. NPY and Y2R Transcript Levels Showed a Region-Selective Decrease in LPS Model Rats

**Figure 4:**
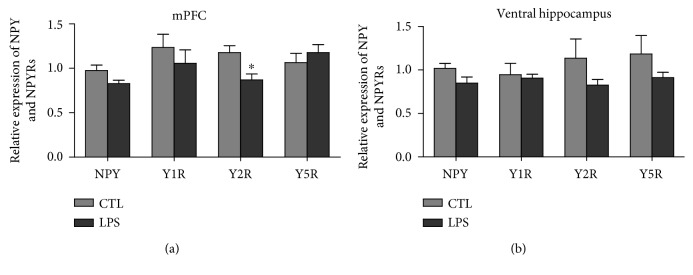
The expression of NPY and Y2R in the mPFC of the LPS group was decreased, while the expression of other NPYRS in the mPFC and ventral hippocampus showed no change. (a) NPY and Y2R showed decreased mRNA expression in the mPFC of the LPS group compared to CTL rats, Y2R: *P* < 0.05, *t*(10) = 3.034, *P* = 0.0104; NPY: *P* < 0.05, *t*(10) = 2.195, *P* = 0.0486. (b) There were no significant differences of Y1R and Y5R expression in the two brain region. CTL: *n* = 6, LPS: *n* = 6. Values were expressed as mean ± SEM. Data were analyzed by independent *t*-test.

Transcript levels of NPY and NPYRS (including Y1R, Y2R, and Y5R) in the mPFC and ventral hippocampus regions from LPS and CTL rats were examined using Q-PCR. The mRNA expression levels of NPY and Y2R were significantly decreased in the mPFC from LPS rats compared with CTL rats (1.10 ± 0.06 vs. 0.82 ± 0.04 and 1.18 ± 0.08 vs. 0.87 ± 0.07, *P* < 0.05, respectively) (Figure 4(a)). However, no significant difference in the expression levels of NPY and Y2R in the ventral hippocampus was seen between two groups (Figure 4(b)). There were no significant differences in the Y1R and Y5R expressions in these two brain regions between the LPS group and the CTL group (Figures 4(a) and 4(b)).

### 3.4. Injection of NPY or PYY (3-36) into the mPFC Reversed the LPS-Induced Depressive-Like Behaviors

**Figure 5:**
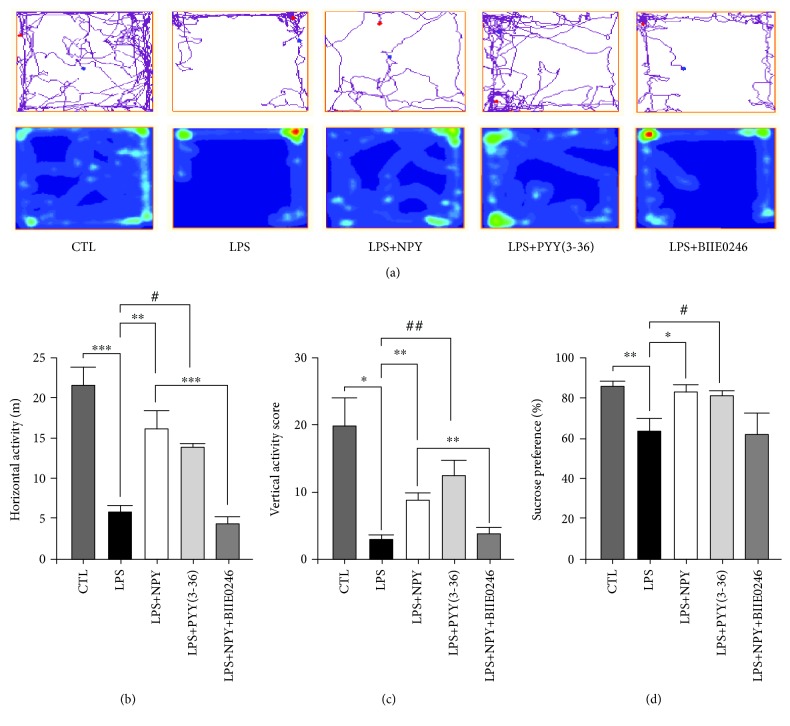
Injection of NPY or PPY (3-36) into the mPFC reversed the LPS-induced depression-like behaviors. (a) The locomotion track of five groups. (b) NPY and PPY (3-36) reversed the LPS-induced decrease of horizontal activity score; BIIE0246 prevented this antidepressant phenomenon of NPY. One-way ANOVA result was *F*(4, 31) = 16.931, *P* < 0.001; (c) NPY or PPY (3-36) treatment prevented the LPS-induced lower vertical activity score; BIIE0246 prevented this effect of NPY. One-way ANOVA result was (*F*(4, 31) = 5.6, *P* = 0.002); (d) NPY or PPY (3-36) treatment prevented the LPS-induced decreased consumption of sucrose in SPT; one-way ANOVA result of SPT was *F*(4, 31) = 4.209, *P* = 0.009. CTL: *n* = 9, LPS: *n* = 6, LPS+NPY: *n* = 5, LPS+PYY (3-36): *n* = 6, LPS+NPY+BIIE0246: *n* = 6; values were expressed as mean ± SEM. Data were analyzed by the LSD multiple comparison tests followed by one-way ANOVA. ^∗^*P* < 0.05, ^∗∗^*P* < 0.01, and ^∗∗∗^*P* < 0.001 compared with the LPS or LPS+NPY group. ^#^*P* < 0.05, ^##^*P* < 0.01 compared with the LPS+PYY (3-36) group.

To test if NPY and Y2R play antidepressant roles in the LPS model, NPY (1 nmol), PYY (3-36) (Y2R agonist, 1 nmol), and BIIE0246 (Y2R antagonist, 1 nmol) were injected into the mPFC, and depressive-like behaviors were carried out at the last day of LPS injection (Figure 1). In OFT, NPY and PYY (3-36) reversed the LPS-induced decreases of horizontal and vertical activity score. Thus, the LPS+NPY group showed a significantly higher horizontal activity score and vertical activity score compared to the LPS group (15.83 ± 2.49 vs. 5.65 ± 1.02 and 8.60 ± 1.08 vs. 2.60 ± 0.75, *P* < 0.01, respectively) (Figures 5(b) and 5(c)). The LPS+PYY (3-36) group also showed a significantly higher horizontal activity score and vertical activity score compared to the LPS groups (13.57 ± 0.55 vs. 5.64 ± 1.02, *P* < 0.01 and 12.33 ± 2.39 vs. 2.60 ± 0.75, *P* < 0.05, respectively) (Figures 5(b) and 5(c)). While the LPS+NPY+BIIE0246 group showed a significantly lower horizontal activity score and vertical activity score compared to the LPS+NPY groups (4.26 ± 1.01 vs. 15.83 ± 2.49, *P* < 0.001 and 3.50 ± 0.99 vs. 8.60 ± 1.08, *P* < 0.01, respectively) (Figures 5(b) and 5(c)). In sucrose preference test, the LPS+NPY and LPS+PYY (3-36) group showed a significantly higher sucrose consumption compared to the LPS group (82.80 ± 3.92 vs. 63.17 ± 6.39% and 81.83 ± 2.48 vs. 63.17 ± 6.39%, *P* < 0.05, respectively) (Figure 5(d)). However, the LPS+NPY+BIIE0246 group only showed a decreased tendency of sucrose consumption (61.83 ± 10.98 vs. 82.80 ± 3.92%, *P* = 0.13) compared to the LPS+NPY group (Figure 5(d)). All those data suggested that application of NPY into the mPFC has antidepressant effect, mainly through Y2R.

### 3.5. Injection of NPY or PYY (3-36) into the mPFC Reversed the Overactivated NLRP3 Pathway Induced by LPS

**Figure 6:**
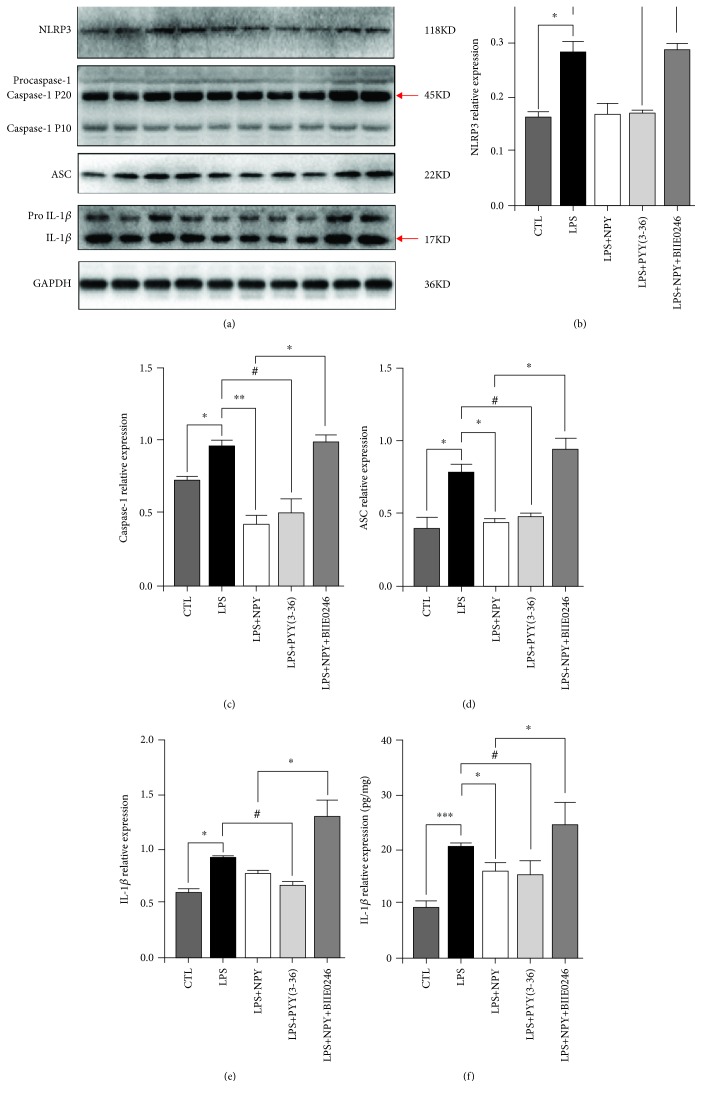
Injection of NPY or PYY (3-36) into the mPFC reversed the LPS-induced overactivation of the NLRP3 pathway. (a) The western blot results of the NLRP3 pathway markers, including NLRP3, caspase-1, ASC, and IL-1*β*. (b) The LPS group showed significantly increased NLRP3 expression, and PYY (3-36) reversed the increased NLRP3 expression of the LPS group; BIIE0246 significantly increased the NLRP3 expression when compared to the NPY+LPS group; one-way ANOVA result was *F*(4, 19) = 47.08, *P* = 0.0001. (c) The LPS group showed significantly increased caspase-1 expression; NPY and PYY (3-36) reversed the increased caspase-1 expression of the LPS group; BIIE0246 significantly increased caspase-1 expression when compared to the NPY+LPS group. One-way ANOVA result was *F*(4, 19) = 55.18, *P* = 0.0001. (d) The LPS group showed significantly increased ASC expression; NPY and PYY (3-36) reversed the increased ASC expression of the LPS group; BIIE0246 significantly increased ASC expression when compared to the NPY+LPS group. One-way ANOVA result was *F*(4, 19) = 43, 68, *P* = 0.0001. (e) The LPS group showed significantly increased IL-1*β* expression, and PYY (3-36) reversed the increased IL-1*β* expression of the LPS group; BIIE0246 significantly increased IL-1*β* expression when compared to the NPY+LPS group. One-way ANOVA result was *F*(4, 19) = 43.74, *P* = 0.0001. (f) The LPS group rats had significantly increased the expression of IL-1*β* detected by ELISA compared to the CTL group; NPY and PYY (3-36) reversed the increased IL-1*β* expression of the LPS group; BIIE0246 significantly increased the IL-1*β* expression when compared to the NPY+LPS group; one-way ANOVA result was *F*(4, 26) = 9.499, *P* = 0.001. (a–f) CTL: *n* = 4, LPS: *n* = 4, LPS+NPY: *n* = 4, LPS+PYY (3-36): *n* = 4, LPS+NPY+BIIE0246: *n* = 4. (e) CTL: *n* = 6, LPS: *n* = 5, LPS+NPY: *n* = 5, LPS+PYY (3-36): *n* = 5, LPS+NPY+BIIE0246: *n* = 6. Values were expressed as mean ± SEM. Data were analyzed by LSD multiple comparison tests followed by one-way ANOVA. ^∗^*P* < 0.05, ^∗∗^*P* < 0.01, and ^∗∗∗^*P* < 0.001 compared with the LPS or LPS+NPY group. ^#^*P* < 0.05, compared with the LPS+PYY (3-36) group.

To explore whether NPY and Y2R play antidepressant roles by inhibiting the NLRP3 pathway in the mPFC region of LPS rats, we examined the protein expression levels of NLRP3, caspase-1, ASC, and IL-1*β* after treatment of NPY or PYY (3-36). PYY (3-36) reversed the LPS-induced increase of NLRP3, caspase-1, ASC, and IL-1*β* levels (Table 4, Figures 6(a)–6(e)). NPY also reversed the LPS-induced increase of caspase-1 and ASC levels while BIIE0246 blocked NPY effects (Table 4, Figures 6(a)–6(e)). Moreover, ELISA results showed that both NPY and PYY (3-36) reversed the LPS-induced upregulation of IL-1*β* level (Table 4, Figure 6(f)). Meanwhile, BIIE0246 blocked NPY effects on the LPS-induced upregulation of IL-1*β* in the mPFC (Table 4, Figure 6(f)). All these data suggested that NPY inhibits the NLRP3 pathway via Y2R.

## 4. Discussion

In the present study, we demonstrated that injection of LPS for 4 days induced depressive-like behaviors. Inflammatory cytokines which collectively polarize the inflammatory M1 phenotype and NLRP3 inflammasome signaling were upregulated accompanied with decreased expression of NPY and Y2R in the mPFC of LPS rats. Moreover, administration of NPY or Y2R agonist PYY (3-36) into the mPFC ameliorated the LPS-induced depressive-like behaviors while inhibited the NLRP3 inflammasome.

Accumulating evidences suggest that central inflammation plays an important role in the development of depression [1–4]. Microglial activation is the principal component of neuroinflammation in the CNS; the function of microglial cells in the pathophysiology of depression has attracted more and more attention. During inflammatory conditions, microglia can be activated with two subtypes, M1 phenotype (proinflammatory) and M2 phenotype (anti-inflammatory) [23, 24]. The predominant subtypes at the injury are M1 phenotype microglia as increased production of CD11b, Iba-1, MHC-II, IL-1*β*, inducible nitric oxide synthase (iNOS), and the activation of the NLRP3 pathway [25]. Conversely, the activated M2 phenotype is characterized by upregulated anti-inflammatory mediators, such as Arginase-1, CD206, and transforming growth factor-*β* (TGF-*β*) [26, 27]. LPS is a powerful immune system activator which has also been used to generate immune stress depression model [28, 29]. In the present study, we found that injection of LPS for 4 days induced depressive-like behaviors and increased levels of IL-1*β* and M1-type microglia activation markers such as CD11b, Iba-1, and MHC-II. Meanwhile, there was no significant difference in the expression of Arginase-1 (M2-type microglia marker) between CTL and LPS rats, suggesting that mainly proinflammatory but not anti-inflammatory was activated in LPS rats. These results were consistent with the earlier studies that the M1 phenotype microglia can be activated by LPS [30–32]. Moreover, our results further verified that neuroinflammation is a contributing factor in the pathophysiology of depression [33–35].

It has been reported that NPY is involved in the pathologic process of depression [36, 37]. The reduction of NPY in the limbic region has been reported in several models of depression including the Flinders Sensitive Line and Fawn Hooded rats as well as chronic mild stress rats [38–40]. Administration of NPY exerts antidepressant-like effects in the olfactory bulbectomized rats and learned helplessness rats [41, 42]. In the present study, we found that the NPY expression was decreased in the mPFC of the inflammation depression rats induced by LPS. Interestingly, while upregulation of inflammatory markers was found in both the mPFC and the ventral hippocampus, downregulation of the NPY expression was only observed in the mPFC, indicating that the regulation of the NPY transcription by LPS is region specific in rats. Moreover, application of NPY in the mPFC reversed the depressive-like behavior in LPS rats, suggesting NPY in the mPFC has antidepressant effect on LPS-induced inflammation depression.

Among five NPY receptor subtypes, Y1R has been shown to mediate NPY-induced antidepressant-like activity in the olfactory bulbectomized rats and the PTSD model rats as well as in the forced swimming test mouse [43–45]. Meanwhile, antagonist of Y5R has antidepressant-like effects in the CMS rat [46, 47]. In the present study, mRNA expression levels of Y1R, Y2R, and Y5R were measured in both the mPFC and the hippocampus of LPS rats; decreased Y2R expression was only found in the mPFC selectively. The mPFC is one of the dominant brain region that mediated stress response; structural and functional changes of the mPFC have been shown associated with emotional disturbances in human depression patients and rodent depression model [48, 49]. It has been reported that Y2R-like immunoreactivity expresses in the mPFC (both Prl and IL) [11]. Moreover, injection of Y2R agonist into the mPFC had a similar effect of NPY, while Y2R antagonist abolished the antidepressant-like effects of NPY. All those results suggest that Y2R in the mPFC is involved in the pathophysiology of depression and mediates NPY antidepressant effects in LPS rats. However, it has been shown that Y2R antagonist has anti-depressant effect in the olfactory bulbectomized rat [50]. Meanwhile, Y2-/- mice exhibited reduced anxiety-related and depression-like behavior [51]. Therefore, Y2R may play different roles in different depression models and different animals.

NLRP3 inflammasome is a multiple protein complex composed of innate immune sensor NLRP3, ASC, and caspase-1. Activation of NLRP3 complex cleave procaspase-1 to mature caspase-1 and generates bioactive IL-1*β* and IL-18 [52, 53]. It has been shown that increased IL-1*β* is only found in PFC after LPS treatment [54]. IL-1*β* treatment elicits depressive-like behaviors, neuroprogression, and inflammation, and IL-1*β* antagonists were suggested to play antidepressant roles in several mental disorders [55]. Moreover, it has been reported that NLRP3 signaling plays a key role in microglial activation and inflammation in LPS-induced depression [56]. In agreement with earlier studies, we also showed that expression levels of proinflammatory factors such as NLRP3, caspase-1, ASC, and IL-1*β* were increased in the mPFC of LPS rats. The LPS-induced upregulations of NLRP3, caspase-1, ASC, and IL-1*β* were reversed by the application of NPY or PYY (3-36), and the inhibitory effects of NPY were blocked by BIIE0246. The anti-inflammation effect of Y2R agonist has also been reported in endotoxemic animals [57]. Taken together, all those implied that NLRP3 inflammasome-related pathways are involved in antidepressant-like activity of NPY mediated by Y2R in the mPFC of LPS rats. However, the mechanisms underlying the immune-modulating and stress-buffering actions of Y2R contributing to the attenuation of behavioral disturbances caused by peripheral immune challenge are complicated and further studies are required.

## 5. Conclusion

NPY played an antidepressant role in the mPFC by suppressing the NLRP3 signaling pathway, mainly via Y2R, in the LPS-induced depression model rats.

## Figures and Tables

**Table 1:** Sequences of the primers used in Q-PCR.

Primer	Forward	Reverse
GAPDH	GACCACCCAGCCCAGCAAGG	TCCCCAGGCCCCTCCTGTTG
NPY	TGGACTGACCCTCGCTCTAT	TGTCTCAGGGCTGGATCTCT
Y1R	ACACGACTCTTCTTCTGGTGCT	TTACTGTCCCTGATTTTGTCCA
Y2R	GCCTGCCATTCACTCTTACC	CAACGATGTCGGTCCAAAG
Y5R	CTGTCGCCATCCAGTAAGGT	TGGAACGCTTGACTCTCATC
CD11b	CTGGGAGATGTGAATGGAG	ACTGATGCTGGCTACTGATG
Iba-1	TTCCTTCTCTATTACCCCTG	GGTGTTCCTTTTCTTCTCTTGC
MHC-II	AGAGACCATCTGGAGACTTG	CATCTGGGGTGTTGTTGGA
Arginase-1	GGTGGATGCTCACACTGACA	GCAAGCCGATGTACACGATG
Caspase-1	CACGAGACCTGTGCGATCAT	CTTGAGGGAACCACTCGGTC
ASC	TGGCTACTGCAACCAGTGTC	CCAGGCTGGAGCAAAGCTAA
NLRP3	AGCTGCTCTTTGAGCCTGAG	TCTGCTAGGCTCTTTGGTGC
IL-1*β*	AAATGCCTCGTGCTGTCTGA	GATTCTTCCCCTTGAGGCCC

**Table 2:** The information of primary antibody.

Antibody	Species	Dilution ratio	Manufacturer
Anti-GAPDH	Mouse source monoclonal antibody	1 : 500	Santa Cruz
Anti-NLRP3	Rabbit source monoclonal antibody	1 : 10000	Abcam
Anti-ASC	Rabbit polyclonal antibody	1 : 500	ImmunoWay
Anticaspase-1	Rabbit polyclonal antibody	1 : 500	Proteintech
Anti-IL1*β*	Polyclonal goat IgG	1 : 500	R&D systems

**Table 3:** The mRNA expression of CD11b, Iba-1, MHC-II, Arginase-1, NLRP3, ASC, caspase-1, and IL-1*β* in the mPFC and ventral hippocampus.

	Markers of M1-type microglia			Markers of M2-type microglia	Markers of NLRP3 pathway			
	CD11b	Iba-1	MHC-II	Arginase-1	NLRP3	ASC	Caspase-1	IL-1*β*
*mPFC*								
CTL	0.70 ± 0.08	1.03 ± 0.16	0.73 ± 0.14	1.01 ± 0.07	0.73 ± 0.10	0.70 ± 0.29	0.88 ± 0.10	0.69 ± 0.06
LPS	1.88 ± 0.34	1.58 ± 0.17	1.76 ± 0.36	1.07 ± 0.19	1.14 ± 0.17	1.29 ± 1.71	1.25 ± 0.13	14.54 ± 3.98

*Ventral hippocampus*								
CTL	0.58 ± 0.11	0.83 ± 0.05	0.68 ± 0.10	0.91 ± 0.07	0.60 ± 0.17	1.24 ± 0.31	0.91 ± 0.10	0.54 ± 0.08
LPS	1.60 ± 0.23	1.47 ± 0.21	1.77 ± 0.35	1.04 ± 0.14	1.07 ± 0.08	1.53 ± 0.06	1.71 ± 0.33	12.51 ± 3.83

**Table 4:** The protein expression of the markers of the NLRP3 pathway in the mPFC.

	Western blot				ELISA (pg/mg)
	NLRP3	ASC	Caspase-1	IL-1*β*	IL-1*β*
CTL	0.16 ± 0.01	0.39 ± 0.09	0.72 ± 0.03	0.60 ± 0.04	9.30 ± 1.29
LPS	0.28 ± 0.02	0.78 ± 0.06	0.96 ± 0.05	0.91 ± 0.01	20.65 ± 0.65
LPS+NPY	0.17 ± 0.02	0.44 ± 0.03	0.42 ± 0.06	0.77 ± 0.03	16.05 ± 1.63
LPS+PYY (3-36)	0.17 ± 0.01	0.48 ± 0.03	0.51 ± 0.09	0.66 ± 0.05	14.98 ± 2.30
LPS+NPY+BIIE0246	0.28 ± 0.01	0.95 ± 0.08	0.90 ± 0.05	1.31 ± 0.15	24.46 ± 4.42

## Data Availability

The data used to support the findings of this study are available from the corresponding authors upon request.
